# Benefits of physical exercise programs toward people with acquired brain injury: A protocol for systematic review and meta-analysis

**DOI:** 10.1097/MD.0000000000028601

**Published:** 2022-02-04

**Authors:** Marta Pérez-Rodríguez, Andrea Gutiérrez-Suárez, Ruben Barakat, Javier Pérez-Tejero

**Affiliations:** a Department of Health and Human Performance, Universidad Politécnica de Madrid, Spain; b Department of Physiotherapy, Medicine and Biomedical Sciences, Faculty of Physiotherapy, Universidad de A Coruña, A Coruña, Spain; c Department of Social Sciences applied to Physical Activity Sport and Leisure, Universidad Politécnica de Madrid, Spain.

**Keywords:** acquired brain injury, health, physical activity, protocol, related quality of life

## Abstract

**Background:**

Exercise has proven to be a tool improving health related quality of life in people with acquired brain injury (ABI) as part of multidisciplinary team during the subacute and chronic phase. While intervention studies and revisions have been increased in recent years, there is no consensus about the type, frequency and variables of control in exercise interventions. Besides, this collective need programs that respond to different functional levels, given the heterogeneity of people with ABI, not only because of the etiology, but also because of the severity differences over their deficits. The aim of this systematic review and meta-analysis is to summarize the evidence regarding the relationship between exercise and health related quality of life in subacute and chronic phase.

**Methods:**

A protocol of systematic review and meta-analysis will examine the benefits of physical exercise (PE) toward people with ABI will be conducted. A comprehensive search will be conducted in the following electronic databases: MEDLINE, The Cochrane Library, CINAHL, SportDiscus, and Web of Science from inception to July 2020. Independent review authors will evaluate the title and abstract for each trial and disagreements will be solved by discussion with a third author if necessary. Standard pairwise meta-analysis, including heterogeneity analysis, subgroup analysis, and sensitivity analysis, will be performed using the Stata software. The quality evaluation of this study will be completed using the Cochrane collaboration risk of bias tool and the risk of bias assessment will be conducted by the World Health Organization grading of recommendations, assessment, development, and evaluation. The review will be reported in accordance to the preferred reporting items for systematic reviews and meta-analyses statement.

**Results and Conclussion:**

This systematic review and meta-analysis protocol will provide an overview regarding the benefits of PE on functioning, social participation and quality of life toward people with ABI. The variability of outcomes across PE from the selected studies will provide important information for future trial designs. Results of the proposed review will inform practice and the design of future clinical trials. This study will summarize all the selected trials aimed at estimating the effectiveness of applying physical activity programs to ABI users.

**Systematic review registration number:** PROSPERO CRD42020191779.

## 1 Introduction

Acquired brain injury (ABI) is a public health condition which causes long-term consequences, being the most common etiologies traumatic brain injury (TBI) and stroke.^[1–3]^ Numerous are the consequences of this condition and due to its heterogeneity in terms of range of deficits over the patients, it requires individualized approaches conduced by interdisciplinary teams.^[4,5]^ In the recent years, sport sciences experts and health providers became more important in implementing fruitful physical activity (PA) programs for this population during subacute^[6,7]^ and chronic phases.^[8]^ Some of the expected effects are the improvements in health related quality of life (HRQoL)^[9,10]^ as well as in physical^[11]^ and cognitive functions.^[12–14]^

The World Health Organization International Classification of Functioning, Disability, and Health (ICF)^[15]^ provides a framework to consider, from an holistic approach, the assessment and management of people with ABI's daily needs. In addition, ICF is commonly used to describe and to identify recovery factors in this population and to design and implement PA programs.^[16–18]^ The World Health Organization indicates that the term PA refers to any bodily movement that requires energy expenditure, as long as the type of PA, duration, frequency, intensity and volume are specified.^[19]^ Thus, the recent review will be included PA and sport interventions in which participants have autonomy to carry out several functional movements and improve their HRQoL. Several reviews have already shown that PA programs got positive outcomes, even better than those who did not incorporate PA during the rehabilitation process; most studies focused on TBI or stroke patients.^[20–22]^ However, regardless of etiology, people with ABI receive rehabilitation in the same type of centers and show similar functional limitations when performing PA. Considering that PA produces similar benefits in improving deficits stemming from ABI, then, users with different conditions can be in the same PA program, establishing specific adaptations according to their personal characteristics and the phase of their condition, either subacute or chronic phase.^[5,23–25]^

The aim of the systematic review and meta-analysis described in this protocol is to determine the benefits of PA programs toward people with ABI among the different ICF domains, answering the following questions: what are the main features of the PA programs for people with ABI in rehabilitation settings? What are the outcomes of those PA programs on health of people with ABI?

## 2 Methods

This systematic review and meta-analysis protocol is prospectively registered in the international prospective register of systematic reviews with CRD42020191779 reference, and developed in accordance with the preferred reporting items for systematic reviews and meta-analysis protocols 2015 statement. The study design will be strictly guided by the Population, Intervention, Comparison, Outcomes and Study (PICOS) framework.

### 2.1 Eligibility criteria

#### 2.1.1 Study design

For this systematic review and meta-analysis, randomized controlled trials will be included and studies with nonrandom intervention group allocation as well as case studies will be rejected.

Adults aged >18 years who meet the criteria for a clinical diagnosis of ABI will be included, regardless of gender, nationality or education level. Mixed etiology studies (eg, TBI and subacute or chronic stage poststroke) will be included. Only studies with participants with ABI in the last year will be included, while studies where participants with advanced dementia or noncontrolled delirium will be excluded.

#### 2.1.2 Setting and time frame

Only trials reporting PA interventions guided by health professionals will be included. The control group will use a variety of different interventions such as usual care or educational treatment, not including PA components in any case. No limitation toward the intensity, duration or exercise setting over the intervention designs will be established.

### 2.2 Information sources

A comprehensive search will be conducted in the following electronic databases: MEDLINE, The Cochrane Library, CINAHL, SportDiscus, and Web of Science (science and social science citation index) from inception to October, 2021. To identify missed articles in the database search, backward citation searching will be conducted by hand-searching the bibliographies of all articles from the full-text review.

### 2.3 Search strategy

A systematic search strategy will be conducted by 2 reviewer authors (MP and AG). It will be carried out using medical subject headings and a broad range of terms and keywords related to PA interventions and ABI individuals.

### 2.4 Study records

#### 2.4.1 Data management

All the retrieved articles will be managed with Endnote X9 (Clarivate Analytics; Philadelphia, PA) and the duplicated studies will be filtered.

#### 2.4.2 Selection process and data collection

The cited 2 reviewer authors (MP and AG) will independently screen titles and abstracts of all retrieved articles. Abstracts that meet the initial screening criteria as well as relevant PICOS information will be retrieved as full-text articles. Disagreements will be solved by discussion with a third reviewer author (JP). In studies including subjects with a variety of conditions, only data pertaining to conditions of interest will be extracted. If the trial was a multiple-arm randomized controlled trial, all relevant experimental intervention groups and control group data will be extracted. In follow-up studies with multiple time points, only data closest to the end of the intervention will be included. Data will be extracted and entered into a standardized recording data extraction form.

#### 2.4.3 Data items

The following information will be extracted with a predesigned data extraction form: author(s), year of publication, country, participant characteristics (sample size, mean age, gender, stage of condition, additional impairments), intervention characteristics (type, frequency, duration), comparators, outcomes (primary and secondary), and adverse events. When necessary, manuscript authors will be contacted for more information to supplement missing data.

### 2.5 Outcomes and prioritization

The primary outcomes will be determined across the ICF domains: “Body Functions and Structures” and “Activities and Participation” measures, all evaluated with standardized assessment tools. Secondary outcomes of interest are type of PA program, exercise duration, and frequency.

### 2.6 Risk of bias in individual studies

The risk of bias for all included studies will be independently assessed by 2 trained independent reviewers (MP and AG) using Cochrane collaboration risk of bias tool which established validity and reliability. Additionally, the grading of recommendations assessment, development and evaluation framework will be used to assess the quality of evidence across studies for each design and health outcome.

### 2.7 Data synthesis

From studies meeting eligibility criteria, statistical analysis toward the sample size, age, intervention and control outcomes, including means (final scores or change score), standard deviations will be conducted. In randomized cross-over trials, size effects will be only extracted at the first cross-over point to avoid contamination with subsequent intervention regimes. The effects on outcomes between postintervention and preintervention will be expressed as mean differences and standardized mean differences when measured with different instruments and scales not comparable as well as to calculate the effect size (Hedges g) of the intervention and their 95% confidence intervals. Heterogeneity among studies will be evaluated using the I^2^ test, the Cochrane Chi square, and the between-study variance using the tau-square. The statistical analysis will be conducted using the Review Manager software (RevMan 5.2; Cochrane Collaboration, Oxford, UK) and Jamovi Project software The jamovi project (2021). jamovi (Version 1.6) [Computer Software]. Retrieved from https://www.jamovi.org.

## 3 Conclusion

More and more studies are evidencing that PA can improve physical, cognitive and emotional deficits caused by ABI and recovering HRQoL.^[20,26]^ The key will be know what the appropriate type of exercise is and how to implement it according to the phase, severity of impairments and contextual factors.^[24]^ This review and meta-analysis will provide an overview regarding the benefits of PA programs on functioning, social participation and quality of life toward people with ABI. Given the breadth of programs shown to have health effects on ABI patients, there is a need to search all programs’ characteristics. Considering the diffuse nature of the etiologies of ABI, the benefits of all types programs across multiple domains of the ICF system needs to be investigated. It is hoped that the results of this study may help to establish the optimal type of program to rehabilitate individuals with ABI and provide reliable evidence for its wide application. Moreover, the variability of outcomes across all selected studies will provide important information for future trial designs leaning on the ICF domains.

## Author contributions

**Conceptualization:** Marta Pérez-Rodríguez.

**Formal analysis:** Andrea Gutiérrez-Suarez.

**Investigation:** Marta Pérez-Rodríguez and Javier Pérez-Tejero.

**Methodology:** Andrea Gutiérrez-Suarez, Ruben Barakat.

**Supervision:** Ruben Barakat, Javier Pérez-Tejero.

**Validation:** Javier Pérez-Tejero.

**Writing – original draft:** Marta Pérez-Rodríguez, Andrea Gutiérrez-Suarez.
